# Impaired Cerebellum to Primary Motor Cortex Associative Plasticity in Parkinson’s Disease and Spinocerebellar Ataxia Type 3

**DOI:** 10.3389/fneur.2017.00445

**Published:** 2017-08-29

**Authors:** Ming-Kuei Lu, Jui-Cheng Chen, Chun-Ming Chen, Jeng-Ren Duann, Ulf Ziemann, Chon-Haw Tsai

**Affiliations:** ^1^Neuroscience Laboratory, Department of Neurology, China Medical University Hospital, Taichung, Taiwan; ^2^School of Medicine, Medical College, China Medical University, Taichung, Taiwan; ^3^Graduate Institute of Biomedical Sciences, China Medical University, Taichung, Taiwan; ^4^Department of Radiology, China Medical University Hospital, Taichung, Taiwan; ^5^Institute of Cognitive Neuroscience, National Central University, Zhongli, Taiwan; ^6^Institute for Neural Computation, University of California San Diego, San Diego, CA, United States; ^7^Department of Neurology and Stroke, Hertie Institute for Clinical Brain Research, Eberhard-Karls-University, Tübingen, Germany

**Keywords:** cerebellar inhibition, motor cortex, paired associative stimulation, Parkinson’s disease, spinocerebellar ataxia type 3

## Abstract

**Background:**

Functional perturbation of the cerebellum (CB)–motor cortex (M1) interactions may underlie pathophysiology of movement disorders, such as Parkinson’s disease (PD) and spinocerebellar ataxia type 3 (SCA3). Recently, M1 motor excitability can be bidirectionally modulated in young subjects by corticocortical paired associative stimulation (PAS) on CB and contralateral M1 with transcranial magnetic stimulation (TMS), probably through the cerebello–dentato–thalamo–cortical (CDTC) circuit. In this study, we investigated the CB to M1-associative plasticity in healthy elderly PD and SCA3.

**Methods:**

Ten right-handed PD patients, nine gene-confirmed SCA3 patients, and 10 age-matched healthy controls (HC) were studied. One hundred and twenty pairs of TMS of the left M1 preceded by right lateral CB TMS at an interstimulus interval of 2 (CB → M1 PAS_2ms_) and 6 ms (CB → M1 PAS_6ms_) were, respectively, applied with at least 1-week interval. M1 excitability was assessed by motor-evoked potential (MEP) amplitude, short-interval intracortical inhibition (SICI), intracortical facilitation (ICF), and cerebellar inhibition (CBI) at the first dorsal interosseous muscle of the right hand before and after the CB → M1 PAS.

**Results:**

The M1 excitability represented by MEP amplitude was significantly facilitated and suppressed in the HC group by CB → M1 PAS_2ms_ and CB → M1 PAS_6ms_, respectively. The bidirectional modulation on MEP amplitude was absent in the PD and SCA3 groups. SICI and the baseline CBI were significantly reduced in the SCA3 group compared to those of the HC group irrespective of the CB → M1 PAS protocols. There was a significant reduction of CBI immediately and 60 min after the CB → M1 PAS protocols in the HC group but not in the patient groups. No significant change of ICF was found.

**Conclusion:**

Corticocortical CB → M1 PAS can induce bidirectional motor cortical plasticity in M1 for healthy aged subjects. The modulation may be independent of the inhibitory neurocircuits, such as SICI and CBI, and the facilitatory mechanism like ICF. Both patients with PD and SCA3 showed impairment of such plasticity, suggesting significant functional perturbation of the CDTC circuit.

## Introduction

The cerebellum (CB) has dense anatomical and functional connections with sensorimotor cortex (1–3). Functional perturbation in these connections may underlie pathophysiology of several movement disorders, such as Parkinson’s disease (PD), dystonia, and spinocerebellar ataxia (4–6). Recently, non-invasive brain stimulation (NBS) has been extensively used as a tool in studying the CB–M1 interactions [for a review, see Tremblay et al. (7)]. For instance, the physiological cerebellar inhibition (CBI) to M1 is currently tested by paired transcranial magnetic stimulation (TMS) of the CB and the contralateral M1 (8). Theta burst stimulation, a type of patterned repetitive transcranial magnetic stimulation (rTMS) (9), of the lateral CB is able to modulate the excitability of the contralateral primary motor cortex (M1) in healthy subjects (10). Paired associative stimulation (PAS) and transcranial direct current stimulation (tDCS) are another two types of NBS. The classic PAS protocol consists of repetitive TMS pairing with median nerve electric stimulation (11). Evidence has shown that simultaneous CB tDCS may abolish the M1 motor plasticity induced by PAS (12). In term of the M1 motor plasticity modulated by the cerebellar afferent information, our previous work has shown that a paired corticocortical TMS protocol targeting the CB and the contralateral M1 can change the M1 excitability by following the spike-timing dependent principle in young healthy subjects (13). Such kind of modulation of M1 plasticity is supposed to depend on a functionally normal cerebello–dentato–thalamo–cortical (CDTC) pathway.

Parkinson’s disease is a common movement disorder, in principle involving the basal ganglia pathology. However, the CDTC pathway may also play an important role on the pathogenesis of parkinsonian tremor (4, 14). Spinocerebellar ataxia type 3 (SCA3) has a primary degeneration of the cerebellar nuclei and a wide spreading dysfunction among CB, brainstem, and basal ganglia (5, 15, 16). This study aims to investigate whether M1 motor plasticity is inducible through the paired associative TMS on the CB and the contralateral M1 for the healthy aged subjects, patients with PD and SCA3. We hypothesized that neurodegenerative disorders involving the CDTC circuit may hamper this kind of M1 plasticity.

## Materials and Methods

### Subjects

In total, 29 right-handed (17) subjects were recruited in this study. All of the 10 PD patients (age, 69.1 ± 9.9 years; three females) fulfilled the UK Brain Bank diagnostic criteria (18). There were nine gene-confirmed SCA3 patients (age, 49.7 ± 15.9 years; six females) (Table 1). All patients were requested to discontinue medications for at least 24 h prior to the two sessions of experiments. Ten healthy subjects were recruited as the control group (age, 65.2 ± 14.3 years; four females). All participants gave their written informed consent before joining the study. They all received a routine brain MRI examination to exclude focal structure lesion. The experimental procedures were in accordance with the latest revision of the Declaration of Helsinki. Approval by the local ethics committee of the China Medical University Hospital was obtained (CMUH103-REC1-015).

**Table 1:** Demographic and clinical characteristics of the Parkinson’s disease (PD) and spinocerebellar ataxia type 3 (SCA3) patients.

PD group (No.)	Age (years)	Sex	Disease duration (years)	Motor/total UPDRS (more affected side)	Hoehn and Yahr stage	Medication (daily dose in mg) (LED)
1	66	F	3	38/55 (L)	2.5	Levodopa 300 (300)
2	70	M	8	48/64 (L)	3	Levodopa 350, trihexyphenidyl 3, amantadine 300, rotigotine 4 (770)
3	67	F	7	18/22 (L)	2	Levodopa 300, biperiden 4, ropinirole PR 4 (380)
4	57	M	6	27/33 (L)	2	Levodopa 300, pramipexole 0.75, amantadine 300 (675)
5	79	F	5	32/45 (L)	2.5	Levodopa 100, amantadine 100, propranolol 20 (200)
6	82	M	5	27/41 (R)	2.5	Levodopa 300, entacapone 600, amantadine 100, propranolol 30 (499)
7	75	M	1	16/31 (R)	2	Levodopa 100 (100)
8	80	M	3	24/30 (L)	2	Levodopa 200 (200)
9	62	M	2	28/40 (R)	2	Levodopa 300, biperiden 3, ropinirole 0.75 (315)
10	53	M	3	15/26 (R)	2	Levodopa 200 (200)

**SCA3 group (No.)**	**Age (years)**	**Sex**	**Disease duration (years)**	**Clinical rating scale for cerebellar function^a^**	**Abnormal CAG repeat number**	**Medication (daily dose in mg) (LED)**

1	47	M	20	13	73	Levodopa 100, amantadine 100, baclofen 20, piracetam 2,400 (200)
2	62	F	3	11	62	Amantadine 150, biperiden 3 (150)
3	43	M	16	21	82	Amantadine 300, trihexyphenidyl 4, tizanidine 9, flunarizine HCl 15 (300)
4	75	F	13	24	66	Alprazolam 0.25 (0)
5	30	F	4	12	76	Tizanidine 3, amantadine 300 (300)
6	59	F	10	22	71	None (0)
7	62	M	2	12	66	Piracetam 2,400 (0)
8	30	F	3	12	76	Amantadine 150 (150)
9	39	F	3	14	74	Levodopa 100, amantadine 100 (200)

*^a^The rating scale was developed by S. Massaquoi and M. Hallett. A higher score represents a more severe cerebellar dysfunction. The maximal score of the scale is 30 [for a detail, see Wessel et al. (19)]*.

*F, female; L, left; LED, l-DOPA equivalent dose (20); M, male; R, right; UPDRS, unified PD rating scale*.

### Procedures

#### Measurement of Motor-Evoked Potential (MEP), Short-Interval Intracortical Inhibition (SICI), Intracortical Facilitation (ICF), and CBI

Subjects were seated on a comfortable reclining chair with both arms relaxed. Cortical excitability of the hand representation of the left primary motor cortex (M1_HAND_) was tested with single-pulse and paired-pulse TMS in blocks of measurements immediately before CB → M1 PAS (baseline, B0) and immediately, 30 and 60 min after CB → M1 PAS (P1, P2, and P3, respectively). The target muscle for the electromyographic recordings was first dorsal interosseus (FDI) of the right hand. The individual resting motor threshold (RMT) and active motor threshold (AMT) were determined over the left M1_HAND_, and AMT was additionally determined over the inion (inion AMT) prior to the baseline recording. The detailed procedure for determining RMT and AMT has been described elsewhere (13, 21). SICI, ICF, and CBI were studied using the same paired-pulse TMS protocols as we reported before (13). A double cone coil (inner diameter of each wing, 110 mm; Magstim Co., UK) was used for the cerebellar stimulation. The interstimulus interval of the paired-pulse TMS for SICI, ICF, and CBI was 2, 10, and 6 ms, respectively. These intervals were determined based on the previous studies in which the most significant effect was usually obtained with these intervals (8, 13, 22–24). Due to limitation of the recording time for each session, we did not measure the other intervals in this study. Twenty trials of single MEPs were recorded at each time point (B0, P1, P2, and P3) with the intertrial interval varied ranging from 7.5 to 12.5 s. Monophasic TMS pulse was delivered for the single MEP recording. Twelve single-pulse and 12 paired-pulse TMS with a pseudorandomized order were measured at each time point for SICI, ICF, and CBI. The intertrial interval was ranging from 3.75 to 6.25 s to limit anticipation. Biphasic TMS pulse was used for the SICI, ICF, and CBI recording.

#### CB → M1 PAS

The inion AMT and the TMS intensity, which can evoke around 1 mV peak-to-peak amplitude of the MEP at right FDI muscle, were applied for the CB → M1 PAS. A subthreshold condition TMS pulse with 95% inion AMT was delivered over right CB by using a double cone coil and followed by a suprathreshold M1_HAND_ stimulus with a figure-of-eight coil. There were two intervals between the condition stimulus and the left M1_HAND_ stimulus. Based on the principle of spike-timing dependent plasticity (STDP) and our previous finding, the interval of 6 ms (CB → M1 PAS_6ms_) is supposed to induce LTD-like effect and the interval of 2 ms (CB → M1 PAS_2ms_) to induce LTP-like effect (13). A total of 120 condition-test TMS pairs with monophasic pulse were delivered at a frequency of 0.25 Hz (i.e., 8 min in duration of the CB → M1 PAS) in each of the two sessions. Every subject completed two sessions by a pseudorandomized order with an interval of at least 7 days in order to avoid interactions between the two sessions.

### Statistical Analysis

Data distribution was first examined with Shapiro–Wilk testing (SPSS 16.0). Non-parametric Kruskal–Wallis *H* test and Mann–Whitney *U* test were used for violation of normal distribution. Conditional on a statistical significance of the Kruskal–Wallis *H* test for three-group comparison (*P* < 0.05), *post hoc* analysis was conducted using Mann–Whitney *U* test with Bonferroni’s correction for multiple comparisons. In case of normal distribution, repeated measures analyses of variance were applied to test the effects of CB → M1 PAS on MEP amplitude, SICI, ICF, and CBI. Data are reported as means ± SD if not stated otherwise.

## Results

All of the subjects were cooperative throughout the experimental procedures. None of them reported any noticeable adverse effects during or after the study. The gender distribution was not significantly different between the groups (*P* > 0.1 by Chi-squared test). The mean age of the SCA3 group (49.7 ± 15.9 years) was significantly less than the PD group (69.1 ± 9.9 years; *P* < 0.05 by non-parametric Mann–Whitney *U* test). There were no differences for RMT, AMT, MEP_1mV_ of M1_HAND_, and inion AMT between groups (all *P* > 0.5 by one-way ANOVA, Table 2). The baseline SICI and CBI were significantly less in the SCA3 group (79.1 ± 41.1 and 101.9 ± 17.2%, respectively) than those in the healthy control (HC) group (45.9 ± 22.9 and 84.7 ± 15.1%, respectively) (*P* < 0.05 by Mann–Whitney *U* test, Table 2). The levodopa equivalent dose was higher in the PD group (363.9 ± 220.3 mg) compared to the SCA3 group (144.4 ± 121.0 mg) (*P* < 0.05 by unpaired *t*-test).

**Table 2:** Baseline parameters of transcranial magnetic stimulation (TMS) in the three groups.

	RMT (%MSO)	AMT (%MSO)	MEP_1mV_^a^ (%MSO)	Inion AMT (%MSO)	Conditional TMS intensity for SICI (%MSO)	Baseline SICI^b^(%)	Conditional TMS intensity for ICF (%MSO)	Baseline ICF^b^ (%)	Conditional TMS intensity for CBI (%MSO)	Baseline CBI^b^ (%)
HC	51.6 ± 7.9	47.3 ± 8.1	67.0 ± 12.4	44.1 ± 4.5	40.0 ± 5.5	45.9 ± 22.9^§^	40.0 ± 5.5	148.7 ± 32.7	41.9 ± 4.3	84.7 ± 15.1^§^
PD	48.6 ± 10.4	44.6 ± 9.3	65.1 ± 15.6	42.1 ± 4.5	40.2 ± 8.7	62.4 ± 30.7	39.7 ± 9.5	147.9 ± 41.2	40.0 ± 4.3	91.9 ± 21.6
SCA3	50.9 ± 7.6	47.1 ± 7.2	61.1 ± 13.7	44.1 ± 5.1	42.3 ± 7.5	79.1 ± 41.1^§^	42.3 ± 7.7	146.3 ± 62.7	41.9 ± 4.9	101.9 ± 17.2^§^

*Data are presented as mean ± SD*.

*^a^The intensity of TMS producing motor-evoked potentials (MEPs) of on average 1 mV in peak-to-peak amplitude in the resting first dorsal interosseus*.

*^b^Data shown by the mean conditioned MEP amplitude as a percentage of the unconditioned mean*.

*^§^P < 0.05 by non-parametric Mann–Whitney U test with Bonferroni’s correction*.

*AMT, active motor threshold; CBI, cerebellar inhibition; HC, health control; ICF, intracortical facilitation; MSO, maximum stimulator output; PD, Parkinson’s disease; RMT, resting motor threshold; SCA3, spinocerebellar ataxia type 3; SICI, short-interval intracortical inhibition*.

**Figure 1:**
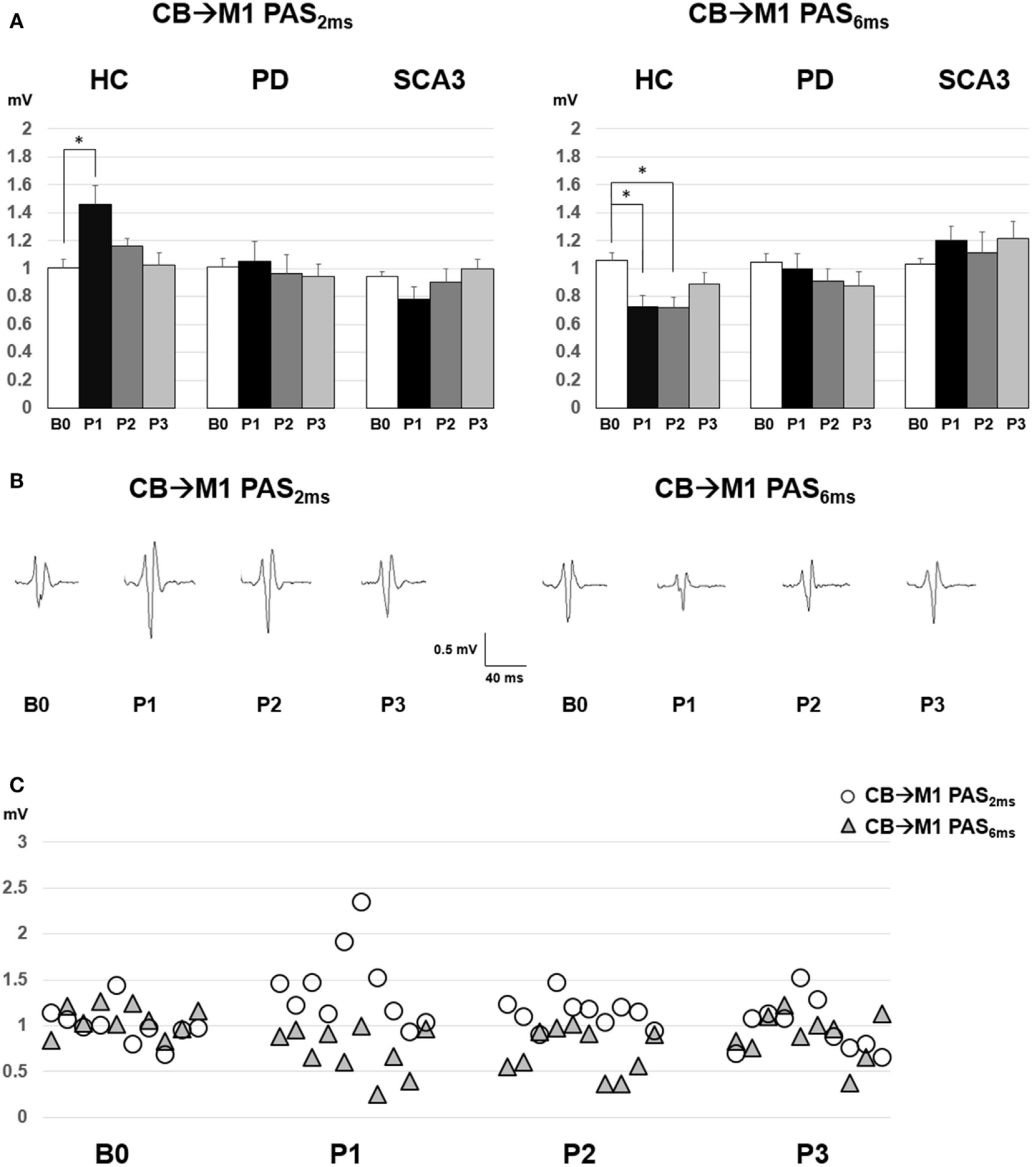
**(A)** Motor-evoked potential (MEP) amplitudes (mean ± SEM in mV) recorded from the right first dorsal interosseus muscle in the healthy control (HC), Parkinson’s disease (PD), and spinocerebellar ataxia 3 (SCA3) groups. Comparisons between pre- (B0), immediate (P1), 30 min (P2), and 60 min (P3) post-CB → M1 PAS_2ms_ vs. CB → M1 PAS_6ms_ were shown. A significant MEP facilitation at P1 after CB → M1 PAS_2ms_ and MEP suppression at P1 and P2 after CB → M1 PAS_6ms_ were noted for the HC group (*P < 0.05 by non-parametric Mann–Whitney *U* test with Bonferroni’s correction). **(B)** The averaged MEP waveforms for the 10 subjects in the HC group. **(C)** Individual MEP data of the HC group (*n* = 10).

**Figure 2:**
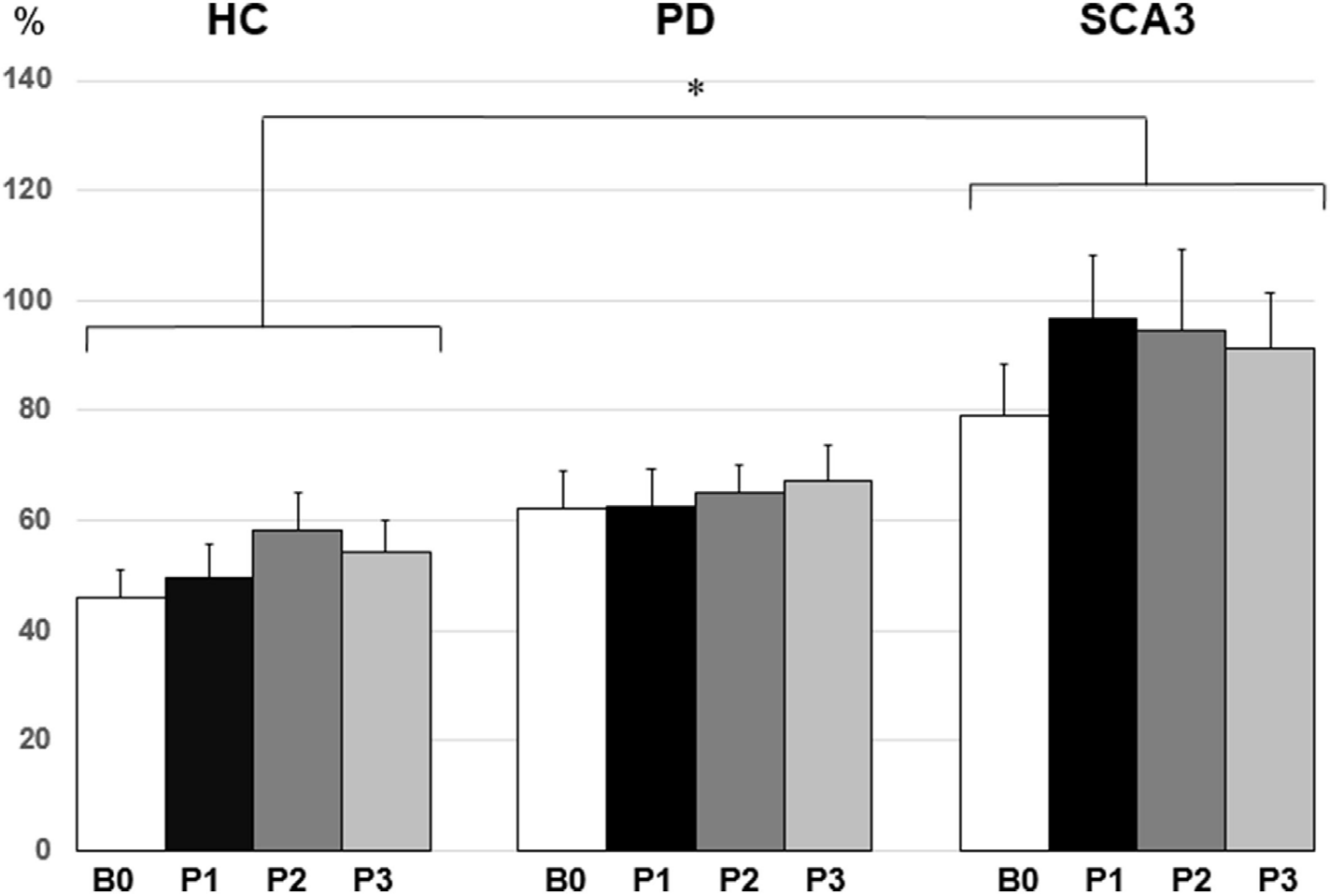
Mean short-interval intracortical inhibition (SICI) [given as percentage of the conditioned motor-evoked potential (MEP)/unconditioned MEP] in the three groups. Note that the spinocerebellar ataxia type 3 (SCA3) group showed a significant reduction of SICI compared to the healthy control (HC) group (*P < 0.05 by non-parametric Mann–Whitney *U* test).

**Figure 3:**
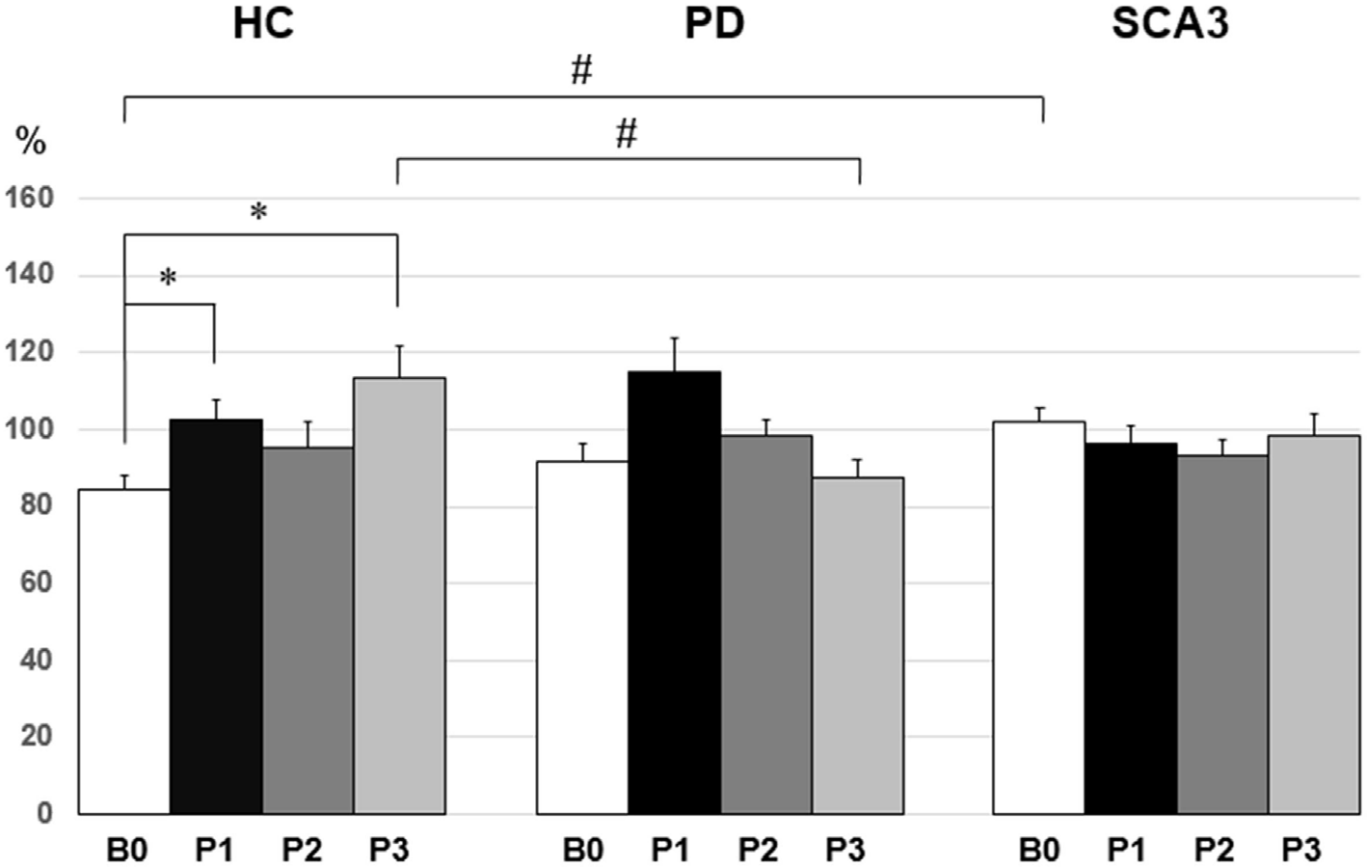
Mean cerebellar inhibition (CBI) [given as percentage of the conditioned motor-evoked potential (MEP)/unconditioned MEP] in the three groups. In the healthy control (HC) group, there was a significant reduction of CBI at P1 and P3 compared to B0 (*P < 0.05 by non-parametric Mann–Whitney *U* test). The spinocerebellar ataxia type 3 (SCA3) group showed a significant reduction of the mean CBI compared with the HC group at B0 (^#^*P* < 0.05 by non-parametric Mann–Whitney *U* test). Compared to the PD group, the HC group showed a significant reduction of the mean CBI at P3 (^#^*P* < 0.05 by non-parametric Mann–Whitney *U* test).

Since violation of normal distribution was found in parts of the MEP, SICI, ICF, and CBI datasets, non-parametric testing was applied to examine the effect of PAS Protocol (CB → M1 PAS_6ms_ vs. CB → M1 PAS_2ms_), Time (B0, P1, P2, and P3), and Group (HC, PD, and SCA3). Comparisons of MEP showed significant MEP facilitation at P1 by CB → M1 PAS_2ms_ and MEP depression at P1 and P2 by CB → M1 PAS_6ms_ for the HC group only (*P* < 0.05 by Mann–Whitney *U* test, Figure 1). Compared to the HC group, a significant reduction of SICI in the SCA3 group was found irrespective of PAS protocols (*P* < 0.05 by Mann–Whitney *U* test, Figure 2). There is a significant reduction of CBI at P1 and P3 compared to B0 in the HC group but not in the other two groups irrespective of PAS protocols (both *P* < 0.05 by Mann–Whitney *U* test, Figure 3). The baseline CBI at B0 was significantly reduced in the SCA3 group compared to the HC group, as also shown in Table 2. In addition, there was a significant difference of CBI at P3 between the HC group and the PD group (Figure 3). The difference was explained by the significant reduction of CBI at P3 in the HC group. There were no effects of PAS Protocol, Time, and Group on ICF (all *P* > 0.05 by Mann–Whitney *U* test).

## Discussion

### CB → M1 PAS-Induced Corticospinal Excitability

The current data revealed that aged subjects have a similar STDP-like M1 motor plasticity induced by CB → M1 PAS as young subjects. Despite aging may decrease the conventional PAS-induced LTP-like plasticity and increase variability (25–27), it might not significantly affect the MEP responses of the CB → M1 PAS. The fact renders a possibility that age may have a distinct influence on different PAS protocols. More data are required to clarify this assumption.

The current data on PD patients are consistent with the findings investigated by conventional PAS. Accumulated evidences have shown that PD patients have a deficit of motor cortical plasticity on their off-medication condition (28, 29). Nevertheless, whether the lack of CB → M1 induced M1 plasticity in our PD patients’ results from M1 pathology or perturbation within the long CDTC pathway is difficult to determine by the current data. Previous TMS studies on tremor have suggested that the CDTC circuit may play a pivotal role in the pathogenesis of parkinsonian tremor (4, 14). Either focal M1 dysplasticity or functional perturbation of the CDTC circuit can disrupt the associative plasticity induced by the CB → M1 PAS.

Patients with cerebellar degeneration also show impaired M1 plasticity by the conventional PAS protocol (30). The conventional PAS provokes STDP-like M1 plasticity through the somatosensory pathway where it has complex connections to the CB. A possible explanation is that M1 plasticity is influenced by the defect of the capability of cerebellar processing of time-specific sensory volleys in these patients (30). The CB → M1 PAS provides a direct route to evaluate whether cerebellar pathology affects the associative plasticity in M1. In this study, we investigated the patients with a homogenous genotype. Findings support the notion that either CB *per se* or its efferent pathways could play a role in M1 motor plasticity.

### SICI/ICF Response

It has been found that the conventional PAS protocol does not alter SICI (11). The effect of the conventional PAS depends on directly antidromic volleys from S1 to M1 neurons where fast-spiking inhibitory interneurons, thought as SICI mediators (31, 32), may not be actively engaged in. Nevertheless, repetitive TMS on CB likely shapes M1 activities through an interaction between these inhibitory interneurons and pyramidal cells (33). The animal study has shown that STDP protocols always result in LTD at synapses between the inhibitory interneurons and pyramidal cells (34). Different from the previous finding showing a non-specific decrease in SICI following CB → M1 PAS protocols (13), SICI was not significantly altered after the CB → M1 PAS in this study. Whether the current CB → M1 protocols modulate GABA-ergic interneurons needs further clarification.

Neurological disorders can diminish or disrupt SICI [for a review, see Berardelli et al. (35)]. Evidences have shown that patients with PD have a reduced SICI (36). Patients with SCA3 also presented a significant reduction of SICI compared to HCs (37). Our data on SICI are consistent with the previous findings and further suggest that patients with SCA3 may bear a more profound functional deficit on M1 inhibitory interneurons compared to patients with PD.

There was no remarkable ICF alteration through the CB → M1 PAS protocols. The finding is consistent with the previous report (13), supporting the notion that distinct mechanisms govern MEP vs. ICF modulation by CB → M1 PAS.

### CBI Response

With the established paired-coil protocol (8), we found that the baseline CBI in the healthy elder subjects was less consistent than the young subjects [84.7 ± 14.9% (Figure 3) vs. 78.4 ± 3.1% (13)]. CBI has been supposed to play an important role in selective tonic muscle movement, probably through a topographically specific reduction of inhibition in M1 (38). The fact that CBI showed a less consistence in the elder subjects might reflex a physiological feature of the aging process in humans.

Cerebellar inhibition was significantly reduced immediately and 60 min after the CB → M1 PAS irrespective of PAS protocol in the control group (Figure 3). It is noted that low-intensity rTMS can modify Purkinje cell dendrites and induce climbing fibers reinnervation (39). It is likely that 0.25 Hz rTMS with the current intensity (i.e., 95% individual inion AMT) actually induce inhibition on the Purkinje cells, which consequently resulted in the reduction of CBI. Nevertheless, CBI did not show any significant change throughout the CB → M1 PAS in the PD group and the SCA3 group. PD patients may bear some degree of functional perturbation in the CDTC pathway (4, 14). Therefore, it would be reasonable that CBI is not well responsive to the CB → M1 PAS which largely depends on a functionally intact CDTC circuit. In addition, CBI may be already impaired in patients with PD (40). The baseline CBI in the current PD group (91.9 ± 21.1%, Figure 3) was consistent with this notion. CBI was not found in our SCA3 group. The finding was compatible with the imaging evidence showing that patients with SCA3 have a significant reduction of the dentate volume and abnormal dentate activation (5).

There are limitations in this study. We only measured the effect of right CB → left M1 PAS. Since the dominant symptoms in our PD patients were not fixed at the same side (Table 1), someone may concern that the inconsistence of the more affected side potentially influences the current finding because motor cortical plasticity induced by conventional PAS has been reported increased on the less affected side and decreased on the more affected side (41). In this study, there were six PD patients presented their more affected side on the left side and four on the right side. Therefore, we would overestimate rather than underestimate the PAS-induced M1 motor plasticity for the PD group. Another concern is the significant difference of the levodopa equivalent dose between the PD and SCA3 group. A higher levodopa equivalent dose was prescribed for the PD group than that for the SCA3 group. Levodopa has been found with a dose-dependent effect to the conventional PAS-induced M1 motor plasticity (42). Despite the fact that we have minimized the confounding factor by ceasing medications for at least 24 h, it can be still difficult to completely exclude the influence caused by the levodopa equivalent dose difference between the PD and the SCA3 group. Nevertheless, the fact that the PD group and the SCA3 group both showed impaired M1 motor plasticity suggests that the difference of the levodopa equivalent dose might be not a key factor influencing the current finding. In this study, the SCA3 group revealed a less mean age than the PD group. People with young age have been found with a more capacity to induce M1 motor plasticity than those with old age (25). Therefore, we would underestimate the impairment of the M1 plasticity for the SCA3 group. Finally, the application of the double cone coil for cerebellar stimulation also bears concerns. The large coil may excite sensory afferent fibers in the brachial plexus or the spinal dorsal nerve roots and antidromically excite the pyramidal tract at the foramen magnum level.

## Conclusion

Corticocortical CB → M1 PAS induced bidirectional motor cortical plasticity in M1 for healthy aged subjects. The modulation relies on the STDP-like principle and seems to be independent of the inhibitory neurocircuits, such as SICI and CBI and the facilitatory mechanism like ICF. There was no such kind of plasticity in patients with PD and SCA3. Findings suggest that neurodegenerative diseases with pathology in the CDTC pathway and/or the CB may erase this type of plasticity. The CB → M1 PAS is supposed to be an alternative route for modulating M1 plasticity and a useful tool in evaluating the functional integrity of the CDTC pathway.

## Ethics Statement

This study was carried out in accordance with the recommendations of the local ethics committee of the China Medical University Hospital with written informed consent from all subjects. All subjects gave written informed consent in accordance with the Declaration of Helsinki. The protocol was approved by the local ethics committee of the China Medical University Hospital (CMUH103-REC1-015).

## Author Contributions

M-KL and J-CC acquired data; M-KL, UZ, and C-HT designed the experiment; C-MC and J-RD analyzed and validated data; C-HT provided research resources; M-KL drafted the original manuscript; All authors reviewed and edited the manuscript.

## Conflict of Interest Statement

The authors declare that the research was conducted in the absence of any commercial or financial relationships that could be construed as a potential conflict of interest. The reviewer, RD, and handling editor declared their shared affiliation and the handling editor states that the process nevertheless met the standards of a fair and objective review.

## References

[1] Allen GI Tsukahara N Cerebrocerebellar communication systems. Physiol Rev (1974) 54(4):957–1006. 4370744 10.1152/physrev.1974.54.4.957

[2] Middleton FA Strick PL. Cerebellar output: motor and cognitive channels. Trends Cogn Sci (1998) 2(9):348–54. 10.1016/S1364-6613(98)01220-0 21227231

[3] Hoover JE Strick PL. The organization of cerebellar and basal ganglia outputs to primary motor cortex as revealed by retrograde transneuronal transport of herpes simplex virus type 1. J Neurosci (1999) 19(4):1446–63. 9952421 10.1523/JNEUROSCI.19-04-01446.1999 PMC6786031

[4] Ni Z Pinto AD Lang AE Chen R. Involvement of the cerebellothalamocortical pathway in Parkinson disease. Ann Neurol (2010) 68(6):816–24. 10.1002/ana.22221 21194152

[5] Stefanescu MR Dohnalek M Maderwald S Thurling M Minnerop M Beck A Structural and functional MRI abnormalities of cerebellar cortex and nuclei in SCA3, SCA6 and Friedreich’s ataxia. Brain (2015) 138(Pt 5):1182–97. 10.1093/brain/awv064 25818870 PMC5963415

[6] Shakkottai VG Batla A Bhatia K Dauer WT Dresel C Niethammer M Current opinions and areas of consensus on the role of the cerebellum in dystonia. Cerebellum (2017) 16(2):577–94. 10.1007/s12311-016-0825-6 27734238 PMC5336511

[7] Tremblay S Austin D Hannah R Rothwell JC. Non-invasive brain stimulation as a tool to study cerebellar-M1 interactions in humans. Cerebellum Ataxias (2016) 3:19. 10.1186/s40673-016-0057-z 27895926 PMC5111316

[8] Ugawa Y Uesaka Y Terao Y Hanajima R Kanazawa I. Magnetic stimulation over the cerebellum in humans. Ann Neurol (1995) 37(6):703–13. 10.1002/ana.410370603 7778843

[9] Huang YZ Edwards MJ Rounis E Bhatia KP Rothwell JC. Theta burst stimulation of the human motor cortex. Neuron (2005) 45(2):201–6. 10.1016/j.neuron.2004.12.033 15664172

[10] Koch G Mori F Marconi B Codeca C Pecchioli C Salerno S Changes in intracortical circuits of the human motor cortex following theta burst stimulation of the lateral cerebellum. Clin Neurophysiol (2008) 119(11):2559–69. 10.1016/j.clinph.2008.08.008 18824403

[11] Stefan K Kunesch E Cohen LG Benecke R Classen J. Induction of plasticity in the human motor cortex by paired associative stimulation. Brain (2000) 123:572–84. 10.1093/brain/123.3.572 10686179

[12] Hamada M Strigaro G Murase N Sadnicka A Galea JM Edwards MJ Cerebellar modulation of human associative plasticity. J Physiol (2012) 590(10):2365–74. 10.1113/jphysiol.2012.230540 22473780 PMC3424758

[13] Lu MK Tsai CH Ziemann U. Cerebellum to motor cortex paired associative stimulation induces bidirectional STDP-like plasticity in human motor cortex. Front Hum Neurosci (2012) 6:260. 10.3389/fnhum.2012.00260 23049508 PMC3446544

[14] Helmich RC Hallett M Deuschl G Toni I Bloem BR. Cerebral causes and consequences of parkinsonian resting tremor: a tale of two circuits? Brain (2012) 135(Pt 11):3206–26. 10.1093/brain/aws023 22382359 PMC3501966

[15] Tokumaru AM Kamakura K Maki T Murayama S Sakata I Kaji T Magnetic resonance imaging findings of Machado-Joseph disease: histopathologic correlation. J Comput Assist Tomogr (2003) 27(2):241–8. 10.1097/00004728-200303000-00023 12703019

[16] Kang JS Klein JC Baudrexel S Deichmann R Nolte D Hilker R. White matter damage is related to ataxia severity in SCA3. J Neurol (2014) 261(2):291–9. 10.1007/s00415-013-7186-6 24272589

[17] Oldfield RC The assessment and analysis of handedness: the Edinburgh inventory. Neuropsychologia (1971) 9(1):97–113. 10.1016/0028-3932(71)90067-4 5146491

[18] Gibb WR Fearnley JM Lees AJ The anatomy and pigmentation of the human substantia nigra in relation to selective neuronal vulnerability. Adv Neurol (1990) 53:31–4. 2173373

[19] Wessel K Zeffiro T Lou JS Toro C Hallett M. Regional cerebral blood flow during a self-paced sequential finger opposition task in patients with cerebellar degeneration. Brain (1995) 118(Pt 2):379–93. 10.1093/brain/118.2.379 7735880

[20] Tomlinson CL Stowe R Patel S Rick C Gray R Clarke CE. Systematic review of levodopa dose equivalency reporting in Parkinson’s disease. Mov Disord (2010) 25(15):2649–53. 10.1002/mds.23429 21069833

[21] Rossini PM Barker AT Berardelli A Caramia MD Caruso G Cracco RQ Non-invasive electrical and magnetic stimulation of the brain, spinal cord and roots: basic principles and procedures for routine clinical application. Report of an IFCN committee. Electroencephalogr Clin Neurophysiol (1994) 91:79–92. 10.1016/0013-4694(94)90029-9 7519144

[22] Kujirai T Caramia MD Rothwell JC Day BL Thompson PD Ferbert A Corticocortical inhibition in human motor cortex. J Physiol (1993) 471:501–19. 10.1113/jphysiol.1993.sp019912 8120818 PMC1143973

[23] Ziemann U Rothwell JC Ridding MC. Interaction between intracortical inhibition and facilitation in human motor cortex. J Physiol (1996) 496(Pt 3):873–81. 10.1113/jphysiol.1996.sp021734 8930851 PMC1160871

[24] Peurala SH Muller-Dahlhaus JF Arai N Ziemann U. Interference of short-interval intracortical inhibition (SICI) and short-interval intracortical facilitation (SICF). Clin Neurophysiol (2008) 119(10):2291–7. 10.1016/j.clinph.2008.05.031 18723394

[25] Fathi D Ueki Y Mima T Koganemaru S Nagamine T Tawfik A Effects of aging on the human motor cortical plasticity studied by paired associative stimulation. Clin Neurophysiol (2010) 121(1):90–3. 10.1016/j.clinph.2009.07.048 19910248

[26] Bhandari A Radhu N Farzan F Mulsant BH Rajji TK Daskalakis ZJ A meta-analysis of the effects of aging on motor cortex neurophysiology assessed by transcranial magnetic stimulation. Clin Neurophysiol (2016) 127(8):2834–45. 10.1016/j.clinph.2016.05.363 27417060 PMC4956500

[27] Lahr J Passmann S List J Vach W Floel A Kloppel S. Effects of different analysis strategies on paired associative stimulation. A pooled data analysis from three research labs. PLoS One (2016) 11(5):e0154880. 10.1371/journal.pone.0154880 27144307 PMC4856316

[28] Udupa K Chen R. Motor cortical plasticity in Parkinson’s disease. Front Neurol (2013) 4:128. 10.3389/fneur.2013.00128 24027555 PMC3761292

[29] Lu MK Chen CM Duann JR Ziemann U Chen JC Chiou SM Investigation of motor cortical plasticity and corticospinal tract diffusion tensor imaging in patients with Parkinsons disease and essential tremor. PLoS One (2016) 11(9):e0162265. 10.1371/journal.pone.0162265 27603204 PMC5014415

[30] Dubbioso R Pellegrino G Antenora A De Michele G Filla A Santoro L The effect of cerebellar degeneration on human sensori-motor plasticity. Brain Stimul (2015) 8(6):1144–50. 10.1016/j.brs.2015.05.012 26140957

[31] Ilic TV Meintzschel F Cleff U Ruge D Kessler KR Ziemann U. Short-interval paired-pulse inhibition and facilitation of human motor cortex: the dimension of stimulus intensity. J Physiol (2002) 545(Pt 1):153–67. 10.1113/jphysiol.2002.030122 12433957 PMC2290644

[32] Di Lazzaro V Pilato F Dileone M Profice P Ranieri F Ricci V Segregating two inhibitory circuits in human motor cortex at the level of GABAA receptor subtypes: a TMS study. Clin Neurophysiol (2007) 118(10):2207–14. 10.1016/j.clinph.2007.07.005 17709293

[33] Casula EP Pellicciari MC Ponzo V Stampanoni Bassi M Veniero D Caltagirone C Cerebellar theta burst stimulation modulates the neural activity of interconnected parietal and motor areas. Sci Rep (2016) 6:36191. 10.1038/srep36191 27796359 PMC5086958

[34] Lu JT Li CY Zhao JP Poo MM Zhang XH. Spike-timing-dependent plasticity of neocortical excitatory synapses on inhibitory interneurons depends on target cell type. J Neurosci (2007) 27(36):9711–20. 10.1523/JNEUROSCI.2513-07.2007 17804631 PMC6672961

[35] Berardelli A Abbruzzese G Chen R Orth M Ridding MC Stinear C Consensus paper on short-interval intracortical inhibition and other transcranial magnetic stimulation intracortical paradigms in movement disorders. Brain Stimul (2008) 1(3):183–91. 10.1016/j.brs.2008.06.005 20633384

[36] Ridding MC Inzelberg R Rothwell JC. Changes in excitability of motor cortical circuitry in patients with Parkinson’s disease. Ann Neurol (1995) 37(2):181–8. 10.1002/ana.410370208 7847860

[37] Farrar MA Vucic S Nicholson G Kiernan MC. Motor cortical dysfunction develops in spinocerebellar ataxia type 3. Clin Neurophysiol (2016) 127(11):3418–24. 10.1016/j.clinph.2016.09.005 27689815

[38] Panyakaew P Cho HJ Srivanitchapoom P Popa T Wu T Hallett M. Cerebellar brain inhibition in the target and surround muscles during voluntary tonic activation. Eur J Neurosci (2016) 43(8):1075–81. 10.1111/ejn.13211 26900871 PMC4836959

[39] Morellini N Grehl S Tang A Rodger J Mariani J Lohof AM What does low-intensity rTMS do to the cerebellum? Cerebellum (2015) 14(1):23–6. 10.1007/s12311-014-0617-9 25346177

[40] Carrillo F Palomar FJ Conde V Diaz-Corrales FJ Porcacchia P Fernandez-Del-Olmo M Study of cerebello-thalamocortical pathway by transcranial magnetic stimulation in Parkinson’s disease. Brain Stimul (2013) 6(4):582–9. 10.1016/j.brs.2012.12.004 23318222

[41] Kojovic M Bologna M Kassavetis P Murase N Palomar FJ Berardelli A Functional reorganization of sensorimotor cortex in early Parkinson disease. Neurology (2012) 78(18):1441–8. 10.1212/WNL.0b013e318253d5dd 22517098 PMC3345788

[42] Fresnoza S Paulus W Nitsche MA Kuo MF. Nonlinear dose-dependent impact of D1 receptor activation on motor cortex plasticity in humans. J Neurosci (2014) 34(7):2744–53. 10.1523/JNEUROSCI.3655-13.2014 24523562 PMC6802752

